# Physiology

**Published:** 1868-03-01

**Authors:** Lemaire J. Paris


### CHICAGO MEDICAL JOURNAL.

*Vol. XXV.*—*MARCH* 1, 1868.—*No.* 5.

### PHYSIOLOGY.

### *Investigations into the nature of Miasmata furnished by the human body in health (read before the Academy of Sciences, the 10th of September, 1867, by Dr. Lemaire J. Paris).*

TRANSLATED EXPRESSLY FOR THE JOURNAL BY WALTER HAY, M.D.

(*Continued from page* 109.)

IN the first part of this work, I think that I proved that the confined air of the barrack and of the casemate, in which I made my experiments, contained a considerable quantity of microphytes and microzoa in process of development.

The extreme neatness of these apartments, and the recent whitewashing of their walls with lime, forbids the supposition that these little beings could have been furnished from dust or dirt accumulated upon the walls or the furniture.

The composition of the vapor of water collected in the exterior atmosphere near these barracks was so different, that one is forcibly led to the conclusion that the soldiers must have furnished them.

This is the object of this communication. I propose, to-day, to demonstrate what portions of the body give origin to them, how they are developed and separated in order to be disseminated throughout the air.

The investigations, which I shall announce, have been made upon men and women from 30 to 70 years of age, and in perfect health.

*Regions of the body where they exist.* In the course of this work, I propose to demonstrate that the organism in action in a physiological state, in perfect health, not only does not furnish microphytes and microzoa, but that it destroys them. It is outside, upon the organs of the skin, and in the mouth that they are found in abundance.

*Skin.* It is known that the bodies of persons who perspire freely are covered rapidly with a deposit composed of different substances, and known under the name of filth (crasse). It is well known, also, that men and women who neglect those cares of the toilet which cleanliness and health demand, sooner or later exhale from certain regions a disagreeable or fœtid odor. The axillary, the ano-perineal, the inguino-scrotal, and the inguino-vulvar regions, the feet, especially between the toes, and the nails are in this condition.

If, after having cleansed these regions with the greatest care with the aid of a solution of saponine, perspiration is excited, and six hours afterwards the sweat is collected and examined with a microscope, there will be found, independently of little fragments of epithelium and of fatty matters, bodies spherical, ovoid, and cylindrical, similar to those whose existence I determined in the confined air of the barrack and the casemate. If, in summer, the washing of the above mentioned parts of men and women be neglected during forty-eight hours, and if perspiration be excited so that a little sweat may be collected, there may be established, except under the axillæ, the presence of bacteria termo and puncta, and of little vibriones. If the bathing of the same regions be neglected during fifteen days, the pasty substance which collects then exhales, about the feet and the genital organs, a fœtid odor. It reddens litmus paper faintly. In this condition, if perspiration be provoked and this matter be examined with the microscope, the existence of the following bodies may be determined: epithelium, globules of fatty matter, some crystals, diaphanous bodies already spoken of, in great numbers; myriads of bacteria (bacteria termo, bacteria catenula formed of two, three, four, and even five segments); bacteria puncta; otick or wand-like vibriones of different dimensions, little spirilla-volutantes and ovoid monads of which some were excavated.

The fœtid dirt packed under the nails of the big toes, diffused in a little distilled water, contained bacteria and little vibriones.

The matter collected during fifteen days under the armpits, contained numerous ovoid spores and only a few bacteria-termo. It reddens litmus paper.

The dirt taken upon the anterior portion of the chest, the epigastrium, the abdomen, and the lumbar and dorsal regions reddened litmus paper strongly. I diffused it in a little distilled water, and found in it numerous diaphanous bodies, ovoid, spherical and cylindrical; also many round spores exhibiting a central nucleus, which made them resemble a piece of money; others ovoid, of which a certain number budded. Some were bi-lobed. The round spores were four to five ten thousandths of a millimetre in diameter. The dimensions of the ovoid spores varied from thirty-five to forty-five ten thousandths of a millimetre in length, and from twenty-five to thirty-five ten thousandths of a millimetre in breadth. I found there no animalcule, which appeared to me due to the strong acidity of the dirt. In the cerumen I found neither spores nor animalculæ.

These experiments are interesting, not only because they demonstrate upon the skin the existence, in great numbers, of the same diaphonous bodies, the same microphytes and microzoa, which I found in the vapor of water collected in the barrack and the casemate, but also on account of the difference which the different regions of the body present in relation to the production of species. The absence of microzoa in the dirt collected upon the skin of the trunk appeared to me to explain the difference of odor between these regions and those where the animalculæ were developed.

These results confirm those which I obtained in my experiments upon ferments, in order to demonstrate that the order of appearance of infusoria is dependent upon the chemical composition of substances. This influence is so great, that I have been able to come to the production of microphytes where microzoa ordinarily are developed, and reciprocally of these latter in place of microphytes, by adding or removing one or several chemical bodies from a natural substance.

The existence of microphytes without microzoa in certain regions, and reciprocally the abundance of microphytes without microzoa in others, appears to me to be dependent on the different chemical composition of their secretions (sweat).

I can not too strongly direct the attention to the influence which the chemical composition of substances exercises upon the development of these little beings, because it appears to me to play the most important part in the history of transmissible maladies.

I shall now proceed to examine how these little beings originate upon the skin.

Physiologists affirm that a man in health, in a state of repose, loses, in twenty-four hours, about a quart of sweat. One who walks or works may lose from four to five quarts daily. Now this sweat, independently of the salts, of the fatty and the lactic acids, contains a perceptible proportion of coagulable albuminoid material. All these bodies form upon the skin a deposit, which accumulates every day. It is increased by the deposition of atmospneric dust, and of that which the clothing always contains. This dirt (crasse) is maintained in a humid state by the insensible perspiration and the sweat. It is in contact with atmospheric air and constantly submitted to a temperature of + 30° Centigrade (108° 32 Fahr.). All these conditions are most favorable to fermentation, and consequently to the development of microphytes and microzoa.

If a laboring man should secrete in twenty-four hours four or five quarts of sweat, his skin will then have received at the end of fifteen the residuum of sixty to seventy-five quarts of sweat, to which must be added the dust of the air and of the clothing. If one reflects upon the quantity of infusoria which might be contained in seventy-five quarts of a liquid holding in solution albuminoid matter, it will occasion no surprise that I have found in this matter myriads of microphytes and microzoa. This is not all. The temperature of the confined air would increase several degrees by reason of the confinement. Moreover, the large quantity of watery vapor furnished at each instant by the lungs and by the cutaneous envelope soon saturates a limited atmosphere. Then the skin remains moist, often even covered with sweat.

All these conditions, doubtless, are very favorable to the development of these little beings, not only upon the skin, but also in the atmosphere in which their reproductive bodies are maintained by reason of their tenuity.

The miasmata which are disengaged from marshes, in warm countries, in which the temperature approximates to that of the human body, exercise a more prompt, and much more violent action than those which are developed in more temperate climates.

I have made experiments in the open air, at Paris, when the thermometer indicated +35° or even +36° Centigrade in the shade. Food, albuminous solutions and other alimentary matters entered rapidly into a state of fermentation. Twelve hours sufficed in order to develop in them bacteria and vibriones. It is therefore incontestable that this elevated temperature renders their reproductive bodies more vigorous, and causes them to arrive more rapidly at the adult state, since at a temperature of 15° to 20° Centigrade at least forty-eight hours is necessary to attain the same result. The elevated temperature of the human body may therefore hasten their development by rendering them more vigorous.

May it not be necessary to refer to this greater vigor, the formidable effects produced by the miasmata evolved from the marshes of hot countries, and by the human body in health? I postpone the consideration of this question; but will, at this time, make use of the fact to explain the presence of animalculæ entirely developed six hours after the condensation of the vapor of water collected in the barrack and in the casemate, as it will equally serve to explain why the vapor of water condensed in the open air, above the fortification, furnished animalculæ only after an interval of forty-eight hours. The temperature was only +16° Centigrade, and their reproductive bodies proceeded from another source.

Micrographs have long since determined that the blood and also the milk removed from the vessels, as well as pus, the remains of the food, and the pultaceous matters which collects upon the teeth, may contain a considerable quantity of micrizoa. The sweat which is supplied at once by the water of the plasma and by the sudoriprous glands, is in the same category as these liquids. Once deposited upon the surface of the body, it ceases to be protected by vitality, and becomes subjected to the common law, *fermentation*. As there is, strictly speaking, no fermentation without infusoria, such appears to me to be the origin of those which I have found in so great numbers upon the skin.

In the experiments whose results I have presented to the Academy, I have demonstrated that the gases and vapors disengaged during alcoholic fermentation and during putrefaction, carry with them into the atmosphere in considerable quantities of germs (propagules) spores and reproductive bodies of microzoa, and even of those little beings entirely developed. It is in this manner that those which exist upon the human skin, appear to me to be carried into the atmosphere.

I would suggest, that, at the present time, both in France and abroad, it is admitted by a great number of physicians that favus, tinea tonsurous, sycosis, and other diseases of the skin are the work of microphytes. These diseases, as is known, are transmissible. In the ages which preceded our own, and even now, physicians acknowledge that uncleanliness is the principal cause of these obstinate maladies. We have just seen that uncleanliness engenders microphytes and microzoa.

Here I am upon the domain of pathology, upon which I do not wish yet to enter. I will mention only, because all these facts are linked together, that I demonstrated at l'hôpital, St. Louis, the existence of spores of the achorion Schonbeinii in a confined atmosphere occupied by patients subjects of favus.

*Mucous Membranes.* The existence of infusoria in the mucus which rests upon these membranes has been indicated. I have collected from several persons, in perfect health, nasal, buccal, pharyngeal, urethral, and vaginal mucus, and bronchial expectoration, and have found in them no infusoria. I have done more—I have preserved this mucus in little bottles corked with emery, in the presence of air, and I have proved that it resisted putrefaction for a much longer time than other organic matters.

These facts agree with those which M. Robin has observed. This skillful observer remarks that organic matters taken into the normal blood, resist putrefaction more than those collected in marshes.

After these facts, I am led to believe that the infusoria which have been found in the mucus have been developed under the influence of a pathological state, or of uncleanliness.

*Mouth.* I have stated that the existence of bacteria and vibriones had been recognized in the remains of the food, and in the pultaceous matters collected upon the teeth. I will add, that persons having carious teeth and irritable gums present moreover, in considerable quantity, spirilla volutantes and monads. There are few adults, otherwise perfectly healthy, who have not some carious teeth, some of this pultaceous matter, and who do not permit some remains of their food to collect between their teeth. It will be understood that those who breathe by the mouth would diffuse through the atmosphere some of these microzoa. It may be demonstrated in the following manner: let the products of respiration passed through the mouth of a man having carious and filthy teeth be directed upon a vessel filled with ice. The vapor of water driven out from the lungs condenses there. There will be found in this liquid, independently of reproductive bodies of microzoa, bacteria and vibrones.

The two bacteria catenala, and the two vibrones which I found in the vapor of water from the casemate, at the moment of the condensation, originated without doubt from the dirty mouths of the soldiers. As I shall demonstrate presently that the vapor of water exhaled from the lungs contains no infusoria, it is therefore, in this case, the mouth alone which could furnish them.

*Products of Expiration.* It is generally admitted that the products of respiration contain organic matters, to which a very prominent part has been assigned in the history of miasmata.

In order to demonstrate their existence, the vapor of water disengaged by the respiratory organs has been condensed, by the aid of cold. This liquid has been left to itself at a temperature of twenty degrees, and some days afterwards it has been observed, that there was formed in it matter which remained suspended; it exhaled the odor of putrefaction. The same experiment has been made upon the air of dissecting rooms, of hospitals, and upon that of marshes. The same phenomena are produced. This matter has been analyzed in all these cases; it contained azote, but a microscopic examination was forgotten.

I have already demonstrated that the matter formed in the vapor of water condensed by this process, over animal matters in a state of putrefaction, and over the marshes of Sologne, is composed of microzoa and microphytes. That which was developed in the vapor of water collected in the barracks also presented a deposit formed of microphytes and microzoa.

Finally, in that which was condensed in the open air, there was produced likewise a deposit, but much less abundant, which may be composed also of microphytes and microzoa, or of microphytes alone, according to the locality. In an experiment which I repeated before M. Chevreul we established this last fact.

This matter, as is seen, is due, in every case, to the development of living beings. When one has reflected that at the moment of condensation the liquid is limpid, and consequently contains no matters in suspension visible to the naked eye; that if it contained either albumenoid matters or fragments of tissue, the one and the other would be fatally destroyed and reduced to their elements by putrefaction, it is probable that one would come to recognize their true nature. In the explanation which is usually given of its formation and its mode of action, this matter ought at once play the part of ferment, decompose itself and develop itself, which is impossible. The demonstration which I have made appears to me to solve this question.

Independent of the facts which I have just reported, and which appeared to me of the highest importance in the history of miasmata, I have established also another which appears to me of no less interest; it is that no deposit is formed in the condensed vapor of water arising from the products of respiration, whilst the contrary is generally believed. Here experimenters have been led into error by their mode of operating.

I have just stated that a deposit, composed of living beings, was formed in the vapor of water condensed from the open air, and that the products of respiration which traverse the mouth carry with them microzoa. The deposit observed in this case is composed of the microphytes and microzoa contained in the mouth and in the air. By observing certain precautions which I shall explain, it may be demonstrated, as I have done in the presence of M. Chevreul, that not only there is no deposit produced, but that there are developed neither microphytes nor microzoa.

Observe how I make this demonstration: I cleanse in advance the entire buccal cavity and the throat with water containing two per cent of tartaric acid, which kills microzoa. I then wash all these parts thoroughly with pure water. I experiment in the morning fasting, in order to avoid the emanations from food, drink, etc., in the following manner: I inspire air through the nostrils and pass the products of expiration through a tube having a bulb surrounded with ice, avoiding carefully the introduction of saliva. For this reason I hold one extremity of the tube between my lips. Twenty minutes suffice to obtain several grammes of condensed vapor. The liquid at the moment of its collection, contains fragments of epithelium, some very small globules, and some black grains, also small; these last appear to me to be carbon. This liquid, placed in a flask stopped with emery, has been examined with the microscope, every two days, in summer, during one month. It has never afforded either microphytes or microzoa, and remained limpid. I repeated this experiment ten times. It has always given me the same results. I have preserved this liquid for a year. It has remained as clear as at the first day.

I proposed to myself the inquiry, if these globules which were found in this liquid might not be the reproductive bodies of infusoria, which could not develop themselves in this liquid, by reason of the absence of food. In order to enlighten myself upon this point I made the two following experiments: To five grammes of this condensed vapor I added fifty centigrammes of albumen obtained from a newly laid egg. I made a comparative experiment with five grammes of distilled water and fifty centigrammes of this same albumen. The two flasks containing about six volumes of air to one of liquid, were shaken, stopped with emery, and left to a temperature which varied from twenty-two to twenty-five degrees Centigrade. I found no difference in the number, nor in the species of animalculæ which were developed in these two liquids, nor in the date of their appearance.

These experiments appeared to me to demonstrate that the products thrown out by the human lungs in health contain neither microphytes nor microzoa.

Now it remains for me to discuss the most difficult point of this question, that is to say, whether these little beings are the cause of typhus, of typhoid fever, of cholera, etc. I have already expressed my opinion upon this subject in 1861. But science demands facts. It is with facts that I shall undertake to solve it, if the Academy will still favor me with its courteous attention. (*To be continued.*)

